# Modeling of lipolysis in the human body and the methodology for developing technology of supplements for obesity prevention considering the utilization of food industry by-products

**DOI:** 10.3389/fnut.2023.1264477

**Published:** 2023-12-08

**Authors:** Vladimir Sadovoy, Nadezhda Barakova, Angelina Baskovtceva, Elena Kiprushkina, Grigory Tochilnikov, Mark Shamtsyan

**Affiliations:** ^1^Department of Commodity Science and Public Catering Technology, Stavropol Institute of Cooperation (Branch), Belgorod University of Cooperation, Economics, and Law, Stavropol, Russia; ^2^Departments of Food Technology and Commodity Science, Institute of Service, Tourism and Design (Branch), North-Caucasian Federal University, Pyatigorsk, Russia; ^3^Faculty of Biotechnology, ITMO University, St. Petersburg, Russia; ^4^Department of Microbiological Synthesis Technology, St. Petersburg State Technological Institute (Technical University), St. Petersburg, Russia; ^5^N.N. Petrov National Research Center of Oncology of the Ministry of Health of Russia, St. Petersburg, Russia

**Keywords:** obesity, lipolysis modeling, computational chemistry, molecular docking, pancreatic lipase, bioactive substances

## 1 Introduction

Obesity is a serious global problem, and extensive research is being conducted on this topic. Obesity is a risk factor for the development of various diseases such as diabetes, atherosclerosis, hypertension, etc. (1–5). It is known that as body weight decreases, there are processes in the body that counteract weight loss and promote relapse of the disease. Therefore, despite certain successes in obesity treatment, the priority lies in the development of new comprehensive approaches that target the diverse disorders in the energy metabolism system, aiming not only to reduce body weight but also to prevent disease relapse (6–9).

The modern pharmaceutical market offers a wide range of drugs for weight reduction (10, 11). One commonly used drug is based on orlistat, a specific inhibitor of gastrointestinal lipases. This drug exhibits high lipophilicity and interacts with fat globules. Orlistat covalently binds to the active site of pancreatic and gastric lipases, thereby inactivating them. As a result of inhibiting gastrointestinal lipases, triglycerides cannot enter the bloodstream (12, 13). This creates an energy deficit, leading to the mobilization of fat from the depot. The drug is manufactured by many pharmaceutical companies, such as Polpharma (Poland), Izvarino Pharma (Russia), and KRKA (Slovenia). The most popular supplements for weight loss include conjugated linoleic acid (CLA) and L-carnitine (14, 15).

CLA's mechanism of action involves influencing the lipase enzyme function. This nutrient helps prevent the accumulation of excess adipocyte cells in subcutaneous adipose tissue and accelerates the process of lipolysis (the metabolic process of fat splitting) (16). On the other hand, L-carnitine acts as a carrier for fats, speeding up their metabolism. It does not participate in the fat-splitting reaction or any other reactions. After the fat molecule is split apart inside the cell, the drug is excreted with its structure virtually unchanged. During the breakdown of fat cells, a significant amount of energy, water, and carbon dioxide is released. In other words, it facilitates a natural metabolic process that the drug accelerates (17–20).

## 2 Methods of pancreatic lipase inhibition

### 2.1 The use of computational chemistry and artificial intelligence in modeling and optimizing the lipolysis process

The utilization of computational chemistry and artificial intelligence aims to minimize the need for costly experimentation. It is crucial to accurately perform geometric optimization of the studied dietary supplements and investigate their molecular properties, including dipole moment, RMS gradient, electron density, and potential energy. Analyzing the changes in potential energy, atomic charges, synchronous transition state search, and studying the quantum-chemical characteristics of the lipase ligand will provide insights into the variations in the activity of pancreatic lipase in the presence of different dietary supplements. For this purpose, molecular docking applications are employed (21, 22).

Artificial intelligence (AI) is associated with fuzzy logic methods and proves effective in processing experimental results. Using AI, optimal composition variants can be identified based on multiple parameters when inhibiting pancreatic lipase (23).

### 2.2 Inhibition of fat accumulation by pancreatic lipase enzyme

Scientists from the Department of Pharmacognosy, JSS College of Pharmacy, India, are engaged in similar studies. Their research suggests that inhibiting fat accumulation through pancreatic lipase enzyme inhibition is an interesting approach in the development of new and safer anti-obesity medications. The pancreatic lipase enzyme is naturally lipolytic and is released from the acinar cells of the pancreas, which play a key role in the breakdown of triglycerides. Thus, the pancreatic lipase enzyme plays an important role in the intestine and is not directly absorbed into the bloodstream, thereby avoiding other associated side effects and complications (24, 25).

### 2.3 Flavonoids—inhibitors of the pancreatic lipase enzyme

A significant study conducted by researchers from the University of Applied Sciences Upper Austria focused on analyzing various data on bioactive compounds for lipid metabolism regulation. The study highlighted flavonoids such as quercitrin, isoquercitrin, and afzelin, which were found to reduce triglyceride levels and regulate several adipogenic transcription factors, resulting in a decrease in the expression of proteins associated with lipogenesis. The effects of the flavonoid kaempferol and the flavone hesperidin against obesity were described. The research was conducted on 3T3-L1 mouse cells. Kaempferol not only suppressed adipogenesis in preadipocytes by 62% but also reduced intracellular lipid accumulation in mature adipocytes by 39%, while hesperidin was able to reduce intracellular lipids by 88.1% (26, 27).

Phenolic compounds, such as flavonoids, saponins, alkaloids, and terpenoids, may possess inhibitory effects on the pancreatic lipase enzyme. This information is supported by *in silico* computer modeling, which identifies the interactions between phytochemicals and the specific active binding site of the pancreatic lipase enzyme. The inhibitory action of lipase is mainly influenced by two factors: the molecule's size and the positioning of hydroxyl groups (28–30).

### 2.4 Phenacyl esters of N-phthaloyl amino acids—inhibitors of pancreatic lipase

Veintramuthu Sankara et al., researchers from the Department of Pharmaceutics, PSG College of Pharmacy, India, are investigating the synthesis and biological evaluation of phenacyl esters of N-phthaloyl amino acids as inhibitors of pancreatic lipase using molecular docking. Their *in vitro* and in silico studies have shown the effectiveness of these compounds in inhibiting pancreatic lipase. In silico ADME (absorption, distribution, metabolism, and excretion) studies have suggested that these compounds may possess medicinal properties (31).

### 2.5 Glutathione disulfide and silibinin (A)—inhibitors of pancreatic lipase

A study conducted by a team of scientists from the Department of Applied Physics, University of Granada (UGR), Spain, has identified new natural compounds from milk thistle components that can inhibit pancreatic lipase. The researchers used the DrugBank database for docking calculations, and the most relevant compounds were further evaluated *in vitro*. The data revealed that glutathione disulfide and silibinin (A) inhibit pancreatic lipase. Therefore, these substances can also serve as promising natural compounds in the fight against obesity (32, 33).

### 2.6 Protein hydrolysates—inhibitors of pancreatic lipase

Chinese scientists from the School of Food Science and Engineering, South China University, China, have also conducted important research and identified economically efficient natural lipase inhibitors against obesity. This study demonstrated, for the first time, the production of porcine pancreatic lipase inhibitory peptides from inexpensive sea buckthorn seed flour using response surface methodology. The results reflected the optimal conditions for obtaining the protein hydrolysate from sea buckthorn seeds (pH, temperature, enzyme dosage, etc.). Under these conditions, the protein hydrolysate inhibited lipase activity by approximately 35%. Analysis using LC-MS/MS revealed the presence of 22 arginine-containing peptides in the hydrolysate, six of which were predicted using molecular docking as potential anti-obesity peptides. The inhibitory action of these peptides against pancreatic lipase involved hydrophilic and hydrophobic interactions, hydrogen bonding, and van der Waals interactions (34).

### 2.7 Plant-based substances facilitating weight loss

Studies describing the positive dyslipidemic effect of ginseng seed oil have attracted great interest. Ginseng, with its high level of ginsenosides as bioactive compounds, reduced lipid content *in vitro* in HepG2 cells and rat hepatocytes, as well as *in vivo* in mice fed a high-fat diet (35, 36).

Another plant, the Indian gooseberry (*Phyllanthus emblica L*.), has a beneficial impact on adipogenesis, as reported by Balusamy et al. from the Department of Food Science and Biotechnology, Sejong University. The researchers found a significant reduction in triglyceride accumulation through the suppression of adiponectin-related adipocyte markers (37).

## 3 Discussion

Of particular interest are studies conducted by scientists engaged in discovering compounds that inhibit pancreatic lipase using molecular docking methods. One promising direction is the development of technologies for obtaining preventive biologically active substances from by-products. Unfortunately, at present, this source of raw materials is not fully utilized, despite being low-cost or even cost-free and having a chemical composition comparable to the primary raw materials.

By-products from the winemaking industry, such as grape seeds and grape pomace (37), or by-products of corn processing, such as husks, cobs, and vegetative parts (38), can be effectively used for extracting flavonoids. Flavonoids are the most valuable components in grape pomace. According to various literature sources, the content of flavonoids in grape pomace ranges from 0.15 to 0.88% (39, 40).

Allicin is a compound with low thermal stability. It slowly degrades at room temperature and rapidly during heating processes (41, 42). Therefore, it is necessary to develop a technology that ensures the delivery of allicin to the small intestine in its native state. The compound allicin can be obtained from garlic which is classified as waste and doesn't meet the standard requirements.

Lecithin is essential for the formation of intercellular spaces, and it is a component of cell membranes. As a building material, lecithin participates in the regeneration of damaged cells. There is evidence that dietary intake of lecithin reduces the absorption of lipids in the body. The mechanism of action of lecithin involves its influence on the activity of the lipase enzyme, helping to prevent the excessive accumulation of adipocytes in subcutaneous adipose tissue and slowing down the process of lipolysis (the metabolic process of fat splitting) (43, 44). To obtain lecithin, it is suggested to use waste from soy protein concentrate production—soy micelles or soy molasses.

L-carnitine is a naturally occurring substance that is similar to B-group vitamins. It is synthesized in the human body and is present in the liver and muscle fibers. L-carnitine mobilizes fat deposits in tissues by transporting fatty acids across the cell membrane, allowing them to be burned in the mitochondria to produce ATP (adenosine triphosphate) (45, 46).

The main representatives of polyunsaturated fatty acids are omega-3 (docosahexaenoic acid, alpha-linolenic acid, eicosapentaenoic acid) and omega-6 (arachidonic acid, linoleic acid). Polyunsaturated fats improve the rheological properties of blood, reduce the level of cholesterol deposits on blood vessel walls, protect cellular membrane lipids from oxidation, and lower triglyceride levels (47, 48). New technologies are being developed to produce omega-3 and omega-6 from by-products of the fish industry, which will help reduce the cost of the final products (49).

The studies involving *in vivo* analysis of the lipolysis process and its regulation using computational chemistry techniques are of particular importance (50).

It is of current interest to investigate the mechanism of lipolysis by studying the intermolecular interactions between the biologically active components of dietary supplements and pancreatic lipase and also to develop compositions that promote activation of metabolic processes, reduction of lipolysis, and decreased fat absorption.

The analysis of the lipolysis mechanism is conducted using computational chemistry methods, which involve the modeling of spatial structures of biologically active substances. Geometric optimization, quantum-chemical characteristics, and charge density distribution of the investigated food supplements and pancreatic lipase molecules enable the prediction of the chemical composition of ingredients that contribute to the reduction of fat absorption in the body. The molecular properties of human pancreatic lipase before and after molecular docking can be studied to determine the conditions for complete blockage of the enzyme's active site.

In addition to studying lipolysis, it is possible to develop preparations that activate metabolism, reduce fat absorption in the intestine, and so on. To scientifically justify the compositional formulation and develop the technology of biologically active supplements for the prevention of obesity, it is necessary to employ semi-empirical methods and molecular mechanics techniques. Based on the conducted experimental research, with the aid of statistical neural networks, it is important to scientifically substantiate and develop compositions of food supplements with preventive properties. The obtained compositions should be evaluated using laboratory animals for their qualitative characteristics, biological value, preventive properties, safety, and harmlessness. Based on the results of these studies, recommendations for their use in nutrition should be developed (51).

One of the means to combat obesity is the inhibition of lipase by biologically active substances of plant and animal origin, extracted, including from secondary raw materials. The evaluation of substance action on lipase is recommended to be conducted through the modeling of spatial structures of biologically active substances using computer chemistry methods and determining the quantum-chemical characteristics and charge density of the molecules of the investigated biologically active substances and lipase.

The use of computer chemistry and artificial intelligence will minimize the costly experiment. It is crucial to accurately perform the geometric optimization of the investigated BADs and examine their molecular properties (dipole moment, RMS gradient, electron density, potential energy).

## Author contributions

VS: Investigation, Writing – review & editing. NB: Conceptualization, Investigation, Writing – original draft. AB: Investigation, Writing – original draft. EK: Conceptualization, Writing – review & editing. GT: Conceptualization, Investigation, Writing – original draft. MS: Conceptualization, Writing – review & editing.
